# Non-Specific GH30_7 Endo-β-1,4-xylanase from *Talaromyces leycettanus*

**DOI:** 10.3390/molecules26154614

**Published:** 2021-07-30

**Authors:** Katarína Šuchová, Nikolaj Spodsberg, Kristian B. R. Mørkeberg Krogh, Peter Biely, Vladimír Puchart

**Affiliations:** 1 Institute of Chemistry, Slovak Academy of Sciences, Dúbravská Cesta 9, SK-845 38 Bratislava, Slovakia; Katarina.Suchova@savba.sk (K.Š.); Peter.Biely@savba.sk (P.B.); 2 Novozymes A/S, Biologiens Vej 2, Kongens Lyngby, DK-2800 Lyngby-Taarbæk, Denmark; NSpo@novozymes.com (N.S.); KBK@novozymes.com (K.B.R.M.K.)

**Keywords:** *Talaromyces leycettanus*, glycoside hydrolase family 30, GH30_7 subfamily, non-specific endo-β-1,4-xylanase, substrate specificity

## Abstract

This study describes the catalytic properties of a GH30_7 xylanase produced by the fungus *Talaromyces leycettanus*. The enzyme is an ando-β-1,4-xylanase, showing similar specific activity towards glucuronoxylan, arabinoxylan, and rhodymenan (linear β-1,3-β-1,4-xylan). The heteroxylans are hydrolyzed to a mixture of linear as well as branched β-1,4-xylooligosaccharides that are shorter than the products generated by GH10 and GH11 xylanases. In the rhodymenan hydrolyzate, the linear β-1,4-xylooligosaccharides are accompanied with a series of mixed linkage homologues. Initial hydrolysis of glucuronoxylan resembles the action of other GH30_7 and GH30_8 glucuronoxylanases, resulting in a series of aldouronic acids of a general formula MeGlcA^2^Xyl_n_. Due to the significant non-specific endoxylanase activity of the enzyme, these acidic products are further attacked in the unbranched regions, finally yielding MeGlcA^2^Xyl_2-3_. The accommodation of a substituted xylosyl residue in the −2 subsite also applies in arabinoxylan depolymerization. Moreover, the xylose residue may be arabinosylated at both positions 2 and 3, without negatively affecting the main chain cleavage. The catalytic properties of the enzyme, particularly the great tolerance of the side-chain substituents, make the enzyme attractive for biotechnological applications. The enzyme is also another example of extraordinarily great catalytic diversity among eukaryotic GH30_7 xylanases.

## 1. Introduction

Xylan is one of the most plentiful polysaccharides on the Earth. It is a major hemicellulose of hardwoods and the second most abundant hemicellulose in softwoods. It also forms a substantial portion of cereals and herbaceous plant cell walls. In general, the xylan backbone is composed of 1,4-linked β-D-xylopyranosyl residues (Xyl*p*). They may be decorated with α-D-glucuronic acid, 4-*O*-methyl-α-D-glucuronic acid and α-L-arabinofuranosyl residues. Moreover, the Xyl*p* residues may be acetylated. The pattern of xylan main chain substitution is species, tissue, and season specific; modulates the physico-chemical properties of the polymer; and renders the hemicellulose water soluble by preventing self-association of the xylan chains [1].

Xylan substitution also has a dramatic impact on its enzymatic digestibility. The depolymerization is catalyzed by endo-β-1,4-xylanases (EC 3.2.1.8). Most of the known xylanases belonging to GH10 and GH11 families act on unbranched or scarcely substituted regions of xylan [2]. Highly decorated xylans are attacked by some GH98 and GH5 xylanases capable of hydrolyzing a glycosidic linkage connecting two substituted Xyl*p* residues [3]. Moreover, it seems that these enzymes not only tolerate but require a certain type of decoration, e.g. 3-*O*-arabinosylation, of Xyl*p* residue accommodated in the −1 subsite [4]. There are also xylanases that require decoration with uronic acids for their action. The latter catalysts can be found in the GH30 family, specifically in subfamilies GH30_7 and GH30_8 [5].

A vast majority of prokaryotic GH30_8 members are (Me)GlcA appendage-dependent enzymes called glucuronoxylanases (EC 3.2.1.136), although some non-specific endoxylanases belong to the subfamily too [6]. The glucuronoxylan specificity is determined by a very strong interaction of the enzyme -2b subsite with the glucuronic acids, which results in the generation of a series of aldouronic acids of the general formula (Me)GlcA^2^Xyl_n_ [7,8]. The same acidic products are formed by eukaryotic GH30_7 glucuronoxylanases although the recognition of (Me)GlcA residue is not entirely the same. However, the substrate and product specificity among GH30_7 members are more diverse since the subfamily also encompasses non-specific endo-β-1,4-xylanases, reducing-end xylose-releasing enzymes, and non-reducing end-acting xylobiohydrolases [6]. The rationale for the catalytic diversity among various GH30_7 enzymes is not fully understood. In this work, we studied the catalytic properties of an earlier patented but insufficiently characterized GH30 xylanase from *Talaromyces leycettanus* [9]. The enzyme shows unique properties with regard to tolerance of the main chain substitution and particularly to the decoration of Xyl*p* residue accommodated in the −2 subsite, resulting in an exceptionally high degree of hydrolysis of different types of xylan. The enzyme thus shows a great potential in numerous biotechnological applications.

## 2. Results

### 2.1. Optimum and Stability of the Enzyme 

Optimal conditions for the enzyme action were tested using beechwood glucuronoxylan as a substrate. The enzyme has an acidic pH optimum 3.0–3.5, while at pH values 2.0 and 5.0 it showed about 75 and 60% of activity (Figure S1). Approaching neutral pH values resulted in a rapid loss of enzyme activity, which was below 10% at pH 6.0. This acidophilic nature of the enzyme has already been reported [9]. Although the enzyme is most active at acidic pH, it remains active upon storage in a relatively broad pH range of 3.0–7.0 [9]. In the 15 min enzyme assay, the enzyme showed a sharp temperature optimum of 60 °C. At higher temperatures, the activity dropped (e.g. to 40% at 70 °C). About 50% of the maximum activity was determined at 40 °C; however, at room temperature (23 °C), the enzyme was almost inactive (approximately 1% activity) (Figure S1). A similar temperature profile has been published [9]. We also examined the temperature stability of the enzyme for the first time. At the temperature optimum, 60 °C, the enzyme gradually lost activity, exhibiting about half activity after 3 h. At 50 °C, the enzyme was fully stable for 2 h and then the activity started to decline. At 40 °C, the enzyme retained activity for 3 h, but upon a longer incubation, the activity was reduced (Figure S1).

### 2.2. Substrate Specificity and Mode of Action

Examination of the substrate specificity of *T. leycettanus* xylanase (*Tl*Xyn30A) on various types of xylan showed that the enzyme efficiently depolymerized hardwood glucuronoxylan, cereal arabinoxylan, as well as algal mixed linkage β-1,3-β-1,4-xylan (rhodymenan), with specific activities (12.4, 3.5, and 3.1 U/mg, respectively).

**Figure 1:**
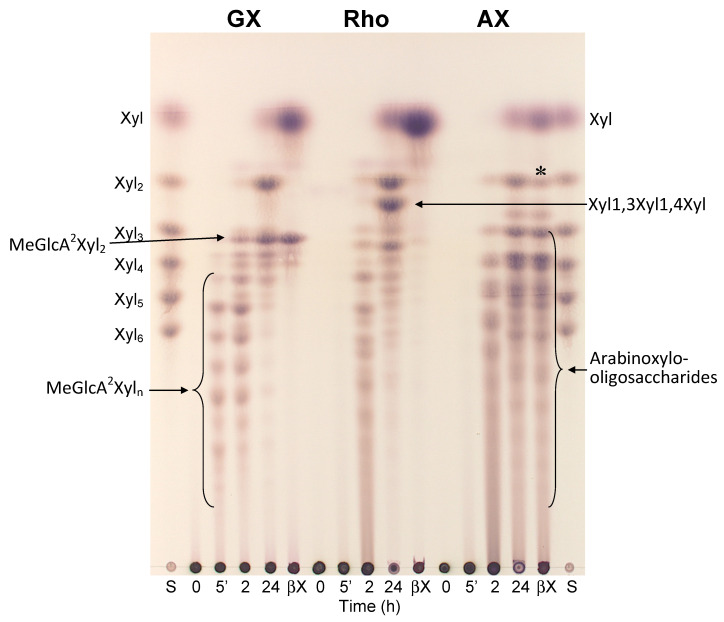
TLC analysis of the products generated by *Tl*Xyn30A xylanase from glucuronoxylan (GX), rhodymenan (Rho), and arabinoxylan (AX). After 24 h, *Tl*Xyn30A was heat-denatured and then the mixtures were treated with β-xylosidase for 24 h (lanes denoted by βX). Note a remarkable change in the products generated from GX and Rho upon the β-xylosidase treatment, in contrast to a resistance of the oligosaccharides generated from AX (marked by an asterisk). S—standards of linear β-1,4-xylooligosaccharides.

**Figure 2:**
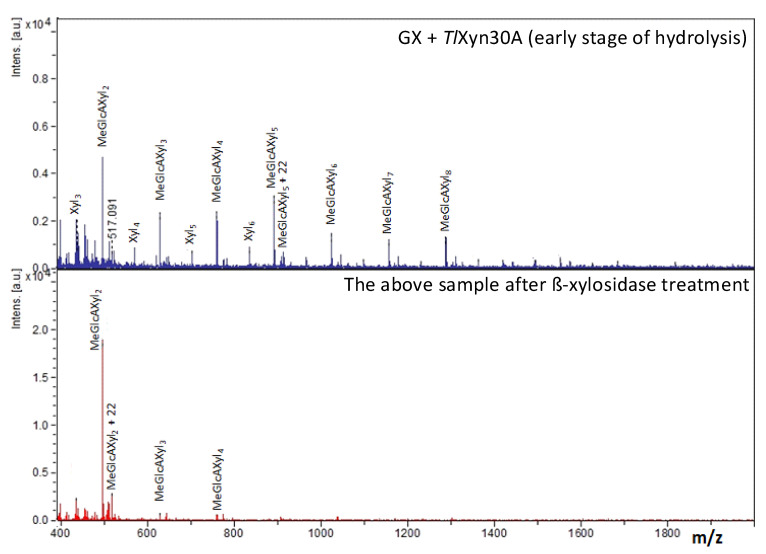
MALDI-ToF MS analysis of early stage reaction products of glucuronoxylan hydrolysis by *Tl*Xyn30A xylanase (upper spectrum), and their conversion to aldotriouronic acid MeGlcA^2^Xyl_2_ accompanied with a disappearance of all linear xylooligosaccharides upon incubation with β-xylosidase (lower spectrum). Prior to addition of β-xylosidase, the *Tl*Xyn30A was thermally denatured. The peaks correspond to sodium adducts of the products. The signals of aldouronic acids are accompanied by ions of their sodium salts (indicated once in each spectrum), which are heavier by an *m*/*z* value of 22.

The hydrolysis products were analyzed by TLC and MALDI-ToF MS. Glucuronoxylan was initially hydrolyzed to a series of products that migrated differently to linear β-1,4-xylooligosaccharides, suggesting that all the products are branched with MeGlcA (Figure 1). Indeed, treatment of an early stage reaction mixture (after a heat-inactivation of *Tl*Xyn30A) with a GH3 β-xylosidase liberating xylose (Xyl) from the non-reducing end resulted in their conversion to a single oligosaccharide, aldotriouronic acid, having a structure MeGlcA^2^Xyl_2_ (Figure 2). This finding serves as evidence that the initial hydrolysis products generated by *Tl*Xyn30A are acidic oligosaccharides of the general structure MeGlcA^2^Xyl_n_, i.e., the fragments have a MeGlcA residue attached to the second Xyl*p* residue from the reducing end.

The aldouronic acids MeGlcA^2^Xyl_n_ did not accumulate in the reaction mixture, but their gradual degradation led to larger linear β-1,4-xylooligosaccharides of a degree of polymerization up to 12, which were further trimmed mainly to xylobiose (Xyl_2_) accompanied by small amounts of Xyl and xylotriose (Xyl_3_). Consequently, the limit hydrolyzate contained a single major aldouronic acid MeGlcA^2^Xyl_2_ with traces of two larger homologs, MeGlcA^2^Xyl_3_ and MeGlcA^2^Xyl_4_. 

TLC monitoring of arabinoxylan hydrolysis indicated that after 5 min, when the acidic products generated from GX were clearly visible on the TLC plate, no detectable oligosaccharides were formed from AX. The initial products of AX hydrolysis were a series of larger arabinoxylooligosaccharides (Figure 1). Upon a longer incubation (24 h), the amount of these products decreased on account of the appearance of a broad palette of shorter products, mostly arabinoxylooligosaccharides resistant towards β-xylosidase. A small amount of Xyl_2_ present in the hydrolysate disappeared after the application of β-xylosidase. 

Rhodymenan (β-1,3-β-1,4-xylan) was hydrolyzed at a rate comparable to AX and was converted to at least two series of oligomeric products. A minor series consists of linear β-1,4-xylooligosaccharides. The series of mixed linkage β-1,3-β-1,4-xylooligosaccharides comprising a single β-1,3-linkage showed a substantially higher chromatographic mobility than β-1,4-xylooligosaccharides. The shortest mixed linkage xylooligosaccharide is an isomeric xylotriose having a structure Xyl*p*β3Xyl*p*β4Xyl, based on our previous publication [5]. After 24 h of incubation, the number as well as the quantity of the larger oligosaccharides was markedly reduced on account of an increase in Xyl, Xyl_2_, and the isomeric xylotriose as the main final products, indicating an ability of *Tl*Xyn30A to hydrolyze certain β-1,3-xylosidic linkages. New faint spots also appeared, which likely correspond to xylooligosaccharides having several β-1,3-linkages. All the products were converted to xylose upon the addition of β-xylosidase (Figure 1).

The enzyme also catalyzed a considerably deeper hydrolysis of acetylglucuronoxylan (not shown) when compared to the performance of other types of endo-β-1,4-xylanases (GH10, GH11, and specific GH30 glucuronoxylanases). This result supported the conclusions obtained with other types of xylan that *Tl*Xyn30A unusually well tolerates xylan side residues. 

### 2.3. Hydrolysis of Short Aldouronic Acids and Linear Xylooligosaccharides

**Figure 3:**
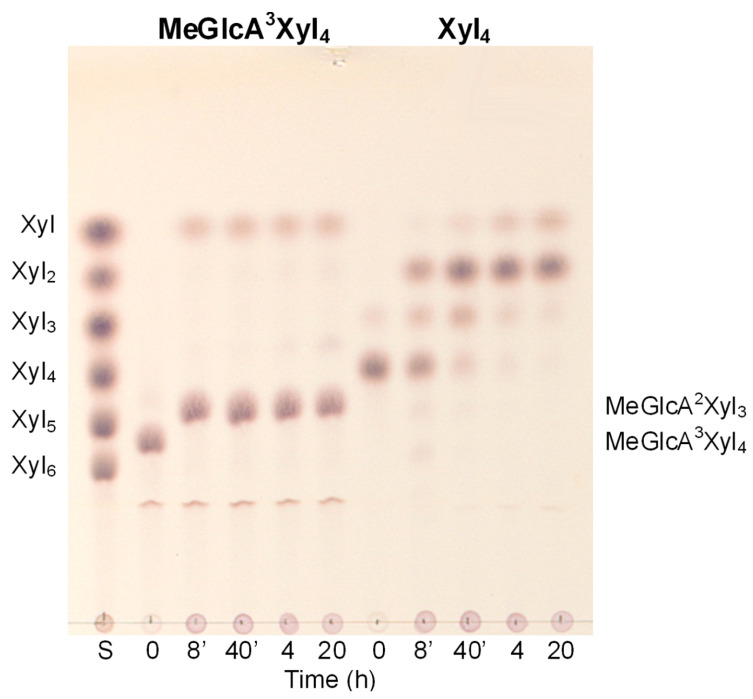
Time course of hydrolysis of aldopentaouronic acid MeGlcA^3^Xyl_4_ and Xyl_4_ by *Tl*Xyn30A xylanase followed by TLC. Notice the difference in the rate of the hydrolysis of the two substrates differing in the MeGlcA side residue. Eight-minute incubation of MeGlcA^3^Xyl_4_ was sufficient for its complete conversion to MeGlcA^2^Xyl_3_, while Xyl_4_ was consumed after several hours. S–standards of linear β-1,4-xylooligosaccharides.

**Figure 4:**
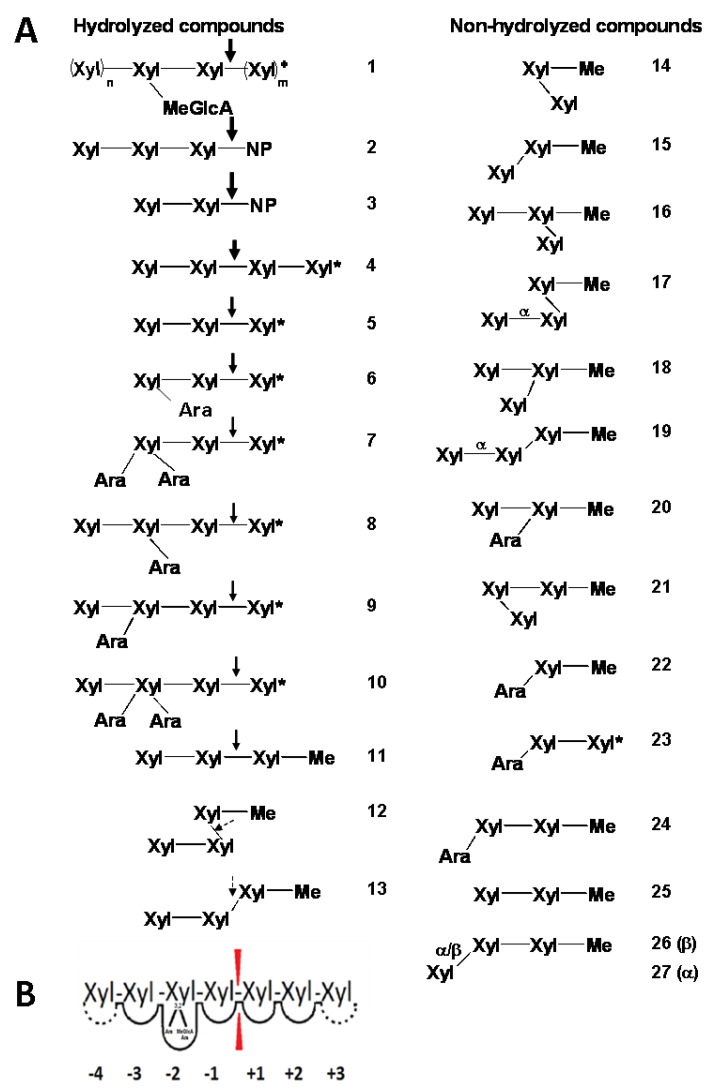
(**A**) The list of oligosaccharides and glycosides (**1**–**27**) tested as substrates for *Tl*Xyn30A and mode of their hydrolysis. The hydrolyzed compounds are in the left column. The arrows of different thickness mark the cleaved linkage and relative hydrolysis rates, with the best substrates on the top. The substituents linked to the xylooligosaccharide main chain with 1,2-glycosidic linkage are facing to the right (e.g., the first compound in the left column, MeGlc^3^AXyl_4_, when m, n = 1) and those linked by 1,3-glycosidic linkage are facing to the left (e.g., the ninth compound in the left column, Xyl4[Ara*f*3]Xyl4Xyl4Xyl). In glycosides, the 4-nitrophenyl and methyl aglycones are abbreviated as NP and Me. The asterisks mark the reducing ends. (**B**) The scheme of the substrate binding site of the enzyme, comprising at least five subsites, indicates that the enzyme tolerates the substituents in the −2 subsite regardless of the position of their attachment.

*Tl*Xyn30A was further examined on naturally occurring low-molecular-weight substrates (Figure 3 and Figure 4). Glucuronoxylan-derived aldouronic acids produced by GH10 and GH11 xylanases, MeGlcA^3^Xyl_3_ (Figure 4A, compound **1**, wherein m = 1 and n = 0) and MeGlcA^3^Xyl_4_ (Figure 4A, compound **1**, wherein m,n = 1), both served as the substrates (starting from this point, the reader is referred to Figure 4A for compound numbering). Both acidic oligosaccharides were shortened by one xylose from the reducing end, yielding MeGlcA^2^Xyl_2_ and MeGlcA^2^Xyl_3_, respectively (Figure 3 and Figure 4A). The cleavage pattern of the short aldouronic acids confirms that the enzyme accommodates MeGlcA-substituted Xyl*p* residue in the −2 subsite.

The hydrolysis rate of the short aldouronic acids was significantly faster than that of unbranched xylooligosaccharides (Figure 3). It is in line with the glucuronoxylanase and endoxylanase activity observed on GX. Among linear xylooligosaccharides, only Xyl_2_ was found to be resistant to the enzyme action, which is consistent with its accumulation in the GX and rhodymenan hydrolyzates. Xyl_3_ (compound **5**) served as a relatively poor substrate, requiring more than 4 h for its entire conversion to an equimolar mixture of Xyl_2_ and Xyl. It is noteworthy that the conversion is not a simple hydrolysis, since larger oligosaccharides, mostly Xyl_5_, were detected in an early stage of the reaction, when a higher trisaccharide concentration was used. The transglycosylation pathway was also observed during Xyl_4_ (compound **4**) degradation, where the initial products were solely Xyl_2_ and Xyl_3_. The tetrasaccharide was consumed within approximately 1 h and the later formation of monomeric Xyl coincided with the disappearance of Xyl_3_. Similarly, Xyl_5_ and Xyl_6_ were initially predominantly converted to an oligosaccharide mixture comprising from disaccharide to the oligosaccharide shorter by one Xyl residue than the substrate. The shortening continued to Xyl_2_ as a final product accompanied with a small amount of Xyl. This mode of action on all linear xylooligosaccharides was not compatible with xylobiohydrolases acting from the non-reducing end and confirms that *Tl*Xyn30A behaves as a retaining endo-β-1,4-xylanase, exhibiting complex reaction pathways that include glycosyl transfer reactions.

### 2.4. Arabinofuranosyl Side Residues Tolerated in the −2 Subsite

We were eager to learn whether arabinosylation at position 2 or 3 of Xyl*p* residue is tolerated by the enzyme. Therefore, singly and doubly decorated xylooligosaccharides arabinosylated on the first or second Xyl*p* residue from the non-reducing end were treated with the enzyme. 3′-Arabinosylated xylobiose (Ara*f*α3Xyl*p*β4Xyl; compound **23**) was left intact (Figure 4A). Quite surprisingly, the enzyme was able to hydrolyze all other tested arabinoxylooligosaccharides comprising at least three xylosyl units (compounds **6**–**10**). All of them were attacked at the second xylosidic linkage from the branch towards the reducing end (Figure 4A), resulting in the liberation of Xyl regardless of the arabinose attached to position 2 and/or 3. A similar accommodation of arabinosylated Xyl*p* residue in the −2 subsite has been reported for a non-specific *Clostridium acetobutylicum* GH30_8 endoxylanase *Ca*Xyn30A [10]. The latter enzyme hydrolyzed 2-*O*-singly and 2,3-doubly arabinosylated xylooligosaccharides faster than 3-arabinosylated, linear, and MeGlcA-substituted counterparts.

### 2.5. Action of TlXyn30A on Artificial Compounds

The enzyme was also tested on several 4-nitrophenyl glycosides. Of them, 4-nitrophenyl glycosides of cellobioside and lactoside were not attacked. In contrast, xylobioside (compound **3**) was readily hydrolyzed, predominantly to Xyl_2_ (Figure 4A), indicating that the aromatic aglycone was bound in the +1 subsite. The most interesting result was obtained with 4-nitrophenyl xylotrioside (compound **2**), forming two productive enzyme-substrate complexes. In the first one, the aromatic aglycone was bound in the +1 subsite (similarly to nitrophenyl xylobioside), resulting in the liberation of the aglycone and Xyl_3_. In the second complex, Xyl_2_ was liberated from the non-reducing end with a simultaneous release of 4-nitrophenyl xyloside. The first cleavage mode was, however, much more frequent (Figure 4A), speaking for endoxylanase action and against xylobiohydrolase activity.

Other compounds examined as the substrates were methyl glycosides of positional isomers of Xyl_2_ and Xyl_3_ (Figure 4A). As expected, β-1,2-xylobioside (compound **14**) as well as β-1,3-xylobioside (compound **15**) did not serve as substrates. β-1,4-Xylobioside (compound **25**) was hydrolyzed extremely slowly to Xyl_2_, which was hardly observed only after 24 h of incubation. β-1,4-Xylotrioside (compound **11**) was hydrolyzed, albeit slowly (about 60% conversion after 4 h) and mainly to methyl xyloside and Xyl_2_. Liberation of methanol and Xyl_3_ was negligible. We further examined the effect of the glycosidic bond between β-1,4-xylobiose and methyl xyloside (Figure 4A). Isomeric linear β-xylotriosides having β-1,2- or β-1,3-linkage at the reducing end (compounds **12** and **13**) were markedly worse substrates (15% conversion after 24 h), although the hydrolysis products were similar to that of orthodox β-1,4-xylotrioside (compound **11**), i.e. β-1,4-xylobiose and methyl xyloside. This finding indicates that *Tl*Xyn30A has a certain ability to hydrolyze β-1,2- as well as β-1,3-xylosidic linkages; however, the natural β-1,4-linkage hydrolysis is clearly preferred.

Other isomeric methyl xylotriosides listed in (Figure 4A) were not hydrolyzed, including methyl β-1,4-xylobiosides in which any of the two xylose residues were substituted (e.g. xylosylated or α-L-arabinosylated) at position 2 or 3.

## 3. Discussion

### 3.1. Catalytic Properties

**Figure 5:**
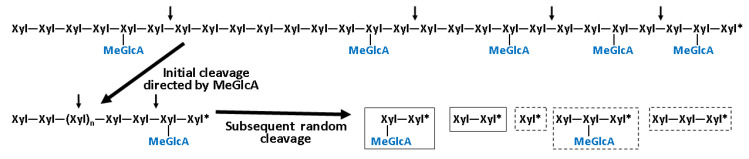
Schematic presentation of the mode of *Tl*Xyn30A action on glucuronoxylan. Initially, the polysaccharide is converted to a mixture of aldouronic acids of a general structure MeGlcA^2^Xyl_n_. Later, the unsubstituted regions are also attacked by the enzyme, yielding aldotriouronic acid MeGlcA^2^Xyl_2_ and Xyl_2_ as major products (in solid boxes) that are accompanied with small amounts of aldotetraouronic acid MeGlcA^2^Xyl_3_, Xyl, and Xyl_3_ (dashed boxes) as limit hydrolysis products.

The studied *Tl*Xyn30A xylanase behaves as a typical endo-acting enzyme, efficiently hydrolyzing several types of xylan. However, hardwood glucuronoxylan is still a preferred substrate since the enzyme recognizes to some extent the MeGlcA side residues. The polysaccharide is initially hydrolyzed to a mixture of aldouronic acids MeGlcA^2^Xyl_n_, suggesting the accommodation of MeGlcA-substituted Xyl*p* residue in the −2 subsite. Such recognition of the MeGlcA side chain was also confirmed by the enzyme action on short aldouronic acids MeGlcA^3^Xyl_3_ and MeGlcA^3^Xyl_4_. This mode of *Tl*Xyn30A initial action is identical to that of other GH30 glucuronoxylanases irrespective of their fungal (GH30_7) or bacterial (GH30_8) origin [6]. However, a distinct feature of *Tl*Xyn30A is that the aldouronic acids are further converted to shorter products and finally to aldotriouronic acid MeGlcA^2^Xyl_2_ with a tiny admixture of aldotetraouronic acid MeGlcA^2^Xyl_3_ (Figure 5). This implies that aglycone-binding subsites +1 and +2 are capable of accommodating MeGlcA-decorated xylose residue. Such shortening of larger aldouronic acids is typical for GH30_7 xylobiohydrolases [5] and some eukaryotic glucuronoxylanases that exhibit xylobiohydrolase activity. The latter may be exemplified by *Talaromyces cellulolyticus* Xyn30B showing minor xylobiohydrolase activity [11] and *Thermothelomyces thermophila* Xyn30A, for which xylobiohydrolase action is roughly equal to glucuronoxylanase activity [12]. These two enzymes yield a roughly equimolar mixture of MeGlcA^2^Xyl_3_ and MeGlcA^2^Xyl_2_ as limit glucuronoxylan hydrolysis products, and the short aldouronic acids are a result of stepwise liberation of Xyl_2_ from the non-reducing end of the initially formed MeGlcA^2^Xyl_n_. This is not the case of the glucuronoxylan hydrolyzate generated by *Tl*Xyn30A, where MeGlcA^2^Xyl_2_ is much more abundant than MeGlcA^2^Xyl_3_. The reason for this difference is that *Tl*Xyn30A exhibits non-specific endoxylanase activity, instead of xylobiohydrolase activity. The non-specific endoxylanase-type mode of action, rather than xylobiohydrolase, is also observed during the cleavage of linear xylooligosaccharides.

The recognition of MeGlcA-substituted Xyl*p* residue in the −2 subsite of *Tl*Xyn30A is not the only substitution that the enzyme can tolerate. Hydrolysis of both 2-*O*- and 3-*O*-singly as well as 2,3-di-*O*-arabinosylated xylooligosaccharides showed that the arabinosylation at these positions does not abolish the enzyme action, and all types of arabinosylated xylose can also be accommodated in the −2 subsite (Figure 4B). Based on the relatively fast chromatographic mobility of the shortest arabinoxylan fragments generated by *Tl*Xyn30A and their resistance to the GH3 β-xylosidases, which is unable to liberate the non-reducing end terminal Xyl*p* residue linked to 3-*O*-arabinoxylated Xyl*p* residue [13], we suggest that the arabinoxylooligosaccharides are of two types with regard of the non-reducing end. Those singly substituted at position 2 are terminated by the 2-*O*-arabinosylated Xyl*p* residue, while those comprising 3-*O*-singly or 2,3-doubly arabinosylated Xyl*p* residue (the vast majority of the fragments) may be terminated at the non-reducing end with an additional non-substituted xylose. Despite the uncertainty about the structure of arabinoxylooligosaccharides, efficient AX degradation to the short fragments is a unique and important capability of the enzyme in view of biotechnological applications.

The mode of cleavage of arabinosylated and glucuronylated xylooligosaccharides showed that a prerequisite for their hydrolysis is the presence of at least two unsubstituted Xyl*p* residues from the branch towards the reducing end. In all cases, the decorated Xyl*p* residue is accommodated in the −2 subsite and the pair of unsubstituted Xyl*p* residues are bound to −1 and +1 subsites. The occupation of the +1 subsite by a short aliphatic aglycone, such as the methyl group, is not sufficient for the formation of a productive enzyme-substrate complex. This fact was documented by the resistance of methyl glycoside of 3′-arabinosylxylobiose (compound **24**) to *Tl*Xyn30A (Figure 4A) and extremely slow hydrolysis of methyl β-1,4-xylobioside (compound **25**). This was in contrast to a single productive binding of methyl β-1,4-trioside (compound **11**), which was more efficiently cleaved to methyl xyloside and Xyl_2_. It is worth mentioning the strong affinity of the +1 subsite for aromatic aglycones, specifically for the 4-nitrophenyl moiety, which results in liberation of the chromophore from 4-nitrophenyl xylobioside and trioside (compounds **3** and **2**) (Figure 4A). In addition, the binding of the aromatic aglycone in the +1 subsite enhances the hydrolysis to such an extent that the aromatic glycosides derived from both linear β-1,4-xylooligosaccharides are hydrolyzed as efficiently as the short aldouronic acids MeGlcA^3^Xyl_3_ and MeGlcA^3^Xyl_4_.

### 3.2. Structure–Function Relationship

*Tl*Xyn30A is composed of an N-terminal catalytic GH30 domain that is linked to a C-terminal CBM1 domain. The presence of the cellulose-binding domain indicates that the enzyme plays a role in the degradation of complex lignocellulose material, wherein hemicellulose xylan is present in close proximity to cellulose. Of the characterized GH30_7 enzymes, *Tc*Xyn30C has a similar architecture [14], although both full-length proteins and catalytic domains are about 50% identical. A similar identity of the catalytic domain was found for other characterized GH30_7 single-domain enzymes, namely *Hypocrea jecorina* glucuronoxylanase XynVI (54.8%) and *Thermothelomyces thermophila* glucuronoxylanase/xylobiohydrolase *Tt*Xyn30A (53.0%). The catalytic domain of *Tl*Xyn30A has the highest homology (up to 75% identity and 85% similarity) to the sequences found in the genomes of several *Aspergillus* species, such as *A. terreus* (Uniprot ID: Q0CUH3), *A. thermomutatus* (A0A397HZ03), *A. turcosus* (A0A421D0F6), *A. udagawae* (A0A0K8KZ21), *A. lentulus* (A0A0S7DIT3), *A. clavatus* (A1C6U9), and *A. novofumigatus* (A0A2I1BUY5), of which the *A. thermomutatus* and *A. clavatus* proteins comprise the CBM1 domain at the C-terminus. Such a high homology suggests that the aforementioned proteins are most probably uncharacterized GH30_7 xylanases that appear to be widespread among *Aspergillus* species.

**Figure 6:**
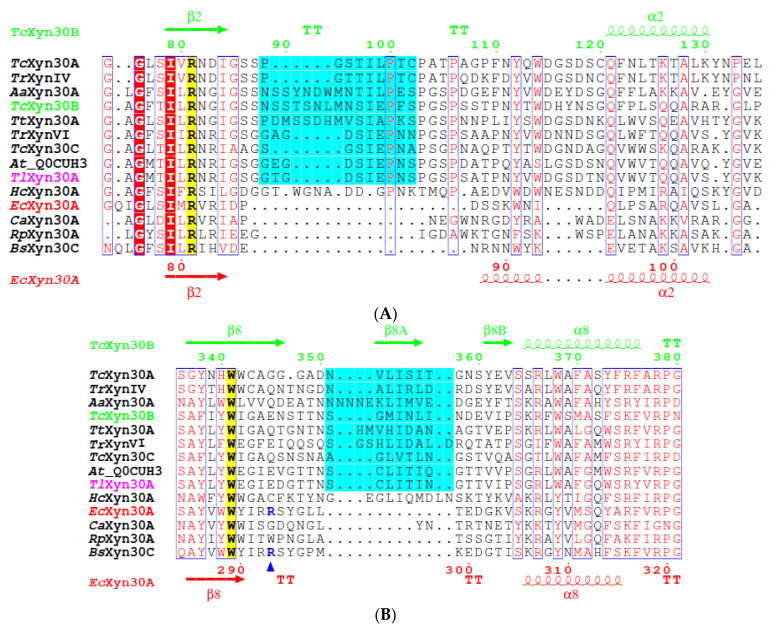
Multiple sequence alignment of *Tl*Xyn30A and selected GH30 xylanases. The first two sequences, *Tc*Xyn30A (Uniprot ID: A0A0B8MZ29) and *Tr*XynIV (A0A024RWW9), represent reducing-end xylose-releasing enzymes. For the next three sequences, *Aa*Xyn30A [5], *Tc*Xyn30B (A0A4V8H018), and *Tt*Xyn30A (G2Q1N4), non-reducing end xylobiohydrolase activity has been demonstrated. The latter two enzymes are specific glucuronoxylanases, similar to *Tr*XynVI (G0RV92). In contrast, *Tc*Xyn30C (A0A6N4SL16) is a non-specific endoxylanase. The next sequence, *At*_QUCUH3, corresponding to an uncharacterized *Aspergillus terreus* protein, shows the highest similarity to *Tl*Xyn30A. The last 5 sequences are of bacterial origin and belong to the GH30_8 subfamily. *Hc*Xyn30A (G8LU16) is a unique prokaryotic non-reducing end xylobiohydrolase. *Ec*Xyn30A (Q46961) and *Bs*XynC (Q45070) are canonical GH30_8 glucuronoxylanases. *Ca*Xyn30A (Q97TI2) and *Rp*Xyn30A (F1TBY8) are exceptional GH30_8 non-specific endoxylanases. Only the regions that are believed to have the highest impact on the unique catalytic properties of *Tl*Xyn30A (typed in violet color) are shown. The corresponding secondary structure elements of *Tc*Xyn30B (green) and *Ec*Xyn30A (red) are shown on the top and bottom. Part (**A**) displays the sequences in the β2-α2 loop, which is considerably larger in eukaryotic GH30_7 enzymes, compared to prokaryotic GH30_8 members. However, even in the eukaryotic sequences, there are differences in the length of the loop that are presumably related to their substrate specificity. Part (**B**) shows the sequences in the β8-α8 region, which contains amino acids interacting with the MeGlcA moiety of the acidic substrates like GX. Among the GX-interacting amino acids is a prokaryotic arginine (typed in blue and marked by an up triangle), which endows the canonical GH30_8 members with glucuronoxylanase specificity. For multiple sequence alignment of entire amino acid sequences, the reader is referred to Supplementary Materials (Figure S2).

All the above mentioned *Aspergillus* proteins as well as *Tl*Xyn30A contain an arginine residue (eukaryotic arginine) that is conserved in the GH30_7 sequences, including *Tc*Xyn30B, for which it has been shown to interact with the carboxyl group of the MeGlcA moiety of the glucuronoxylan-type substrate [15]. The MeGlcA carboxyl group is also hydrogen bonded to *Tc*Xyn30B Glu345 and Ser351 [15]. In the corresponding positions, the same amino acid residues are also found in the sequence of *Tl*Xyn30A (Figure 6B), thus explaining its preference for GX and the mode of its initial cleavage to aldouronic acids MeGlcA^2^Xyl_n_. However, other factors finely tuning the substrate specificity, in particular the xylobiohydrolase activity of the GH30_7 enzymes, are the length and primary structure of the β2-α2 loop in the GH30_7 members. If the loop is long enough, it may interfere with the accommodation of the substituted Xyl*p* residue in the −3 subsite and also obstruct the occupation of more distant negative binding subsites by larger substrates, thus endowing the enzyme with xylobiohydrolase activity [5]. The loop of all the GH30_7 enzymes exhibiting xylobiohydrolase activity, namely *Tc*Xyn30B, *Tt*Xyn30A, and *Aa*Xyn30A, is five amino acids longer than that of *Tl*Xyn30A (Figure 6A) and also the *Aspergillus* proteins of high sequence identity. The shorter β2-α2 loop of *Tl*Xyn30A could be the reason that the loop does not represent any obstacle to bind larger substrates to distant negative subsites, which is a prerequisite for the endoxylanase activity of the enzyme.

## 4. Materials and Methods

### 4.1. Enzymes

Gene cloning and expression as well as encoded protein purification and verification of the purity of *Talaromyces leycettanus* CBS 398.68 GH30 xylanase (55 kDa by SDS PAGE) were described previously [9]. GH3 β-xylosidase is a recombinant *Aspergillus niger* enzyme expressed in *Saccharomyces cerevisiae* [16].

### 4.2. Substrates

Beechwood 4-*O*-methylglucuronoxylan (GX) was prepared as described earlier [17]. Isolation of aspenwood acetylglucuronoxylan by steam explosion was also reported [18]. Rhodymenan, an algal linear β-1,3-β-1,4-xylan from *Palmaria palmata*, was a kind gift of Prof. M. Claeyssens (University of Ghent, Ghent, Belgium). Wheat arabinoxylan (Ara:Xyl 38:62, medium viscosity), 4-nitrophenyl glycosides of xylose, xylobiose (compound **3**) and xylotriose (compound **2**), linear β-1,4-xylooligosaccharides (Xyl_2_-Xyl_6_) and arabinoxylooligosaccharides A3X4X (α-L-Ara*f*-1,3-β-D-Xyl*p*-1,4-D-Xyl; compound **23**), A2X4X4X (α-L-Ara*f*-1,2-β-D-Xyl*p*-1,4-β-D-Xyl*p*-1,4-D-Xyl; compound **6**), A3[A2]X4X4X (α-L-Ara*f*-1,3-[α-L-Ara*f*-1,2]-β-D-Xyl*p*-1,4-β-D-Xyl*p*-1,4-D-Xyl; compound **7**), X4[A3]X4X4X (β-D-Xyl*p*-1,4-[α-L-Ara*f*-1,3]-β-D-DXyl*p*-1,4-β-D-Xyl*p*-1,4-D-Xyl; compound **9**), X4[A2A3]X4X4X (β-D-Xyl*p*-1,4-[α-L-Ara*f*-1,2][α-L-Ara*f*-1,3]-β-D-Xyl*p*-1,4-β-D-Xyl*p*-1,4-D-Xyl; compound **10**) and a mixture of X4[A3]X4X4X (compound **9**) and X4[A2]X4X4X (β-D-Xyl*p*-1,4-[α-L-Ara*f*-1,2]-β-D-Xyl*p*-1,4-β-D-Xyl*p*-1,4-D-Xyl; compound **8**) were purchased from Megazyme International (Bray, Ireland). Xylose was from Serva (Heidelberg, Germany). MeGlcA^3^Xyl_3_ and MeGlcA^3^Xyl_4_ were prepared from beechwood GX as described previously [19]. Methyl glycosides of (arabino)xylooligosaccharides—α-L-Ara*f*-1,3-β-D-Xyl*p*-*O*-Me (A3XMe; compound **22**), β-D-Xyl*p*-1,4-[α-L-Ara*f*-1,3]-β-D-Xyl*p*-*O*-Me (X4[A3]XMe; compound **20**)), α-L-Ara*f*-1,3-β-D-Xyl*p*-1,4-β-D-Xyl*p*-*O*-Me (A3X4XMe; compound **24**)), β-D-Xyl*p*-1,4-β-D-Xyl*p*-*O*-Me (X4XMe; compound **25**)), β-D-Xyl*p*-1,4-β-D-Xyl*p*-1,4-β-D-Xyl*p*-*O*-Me (X4X4XMe; compound **11**)), β-D-Xyl*p*-1,4-β-D-Xyl*p*-1,3-β-D-Xyl*p*-*O*-Me (X4X3XMe; compound **13**)), β-D-Xyl*p*-1,4-β-D-Xyl*p*-1,2-β-D-Xyl*p*-*O*-Me (X4X2XMe; compound **12**)), β-D-Xyl*p*-1,2-β-D-Xyl*p*-1,4-β-D-Xyl*p*-*O*-Me (X2X4XMe; compound **21**)), β-D-Xyl*p*-1,3-β-D-Xyl*p*-*O*-Me (X3XMe; compound **15**)), β-D-Xyl*p*-1,2-β-D-Xyl*p*-*O*-Me (X2XMe; compound **14**)), β-D-Xyl*p*-1,4-[β-D-Xyl*p*-1,2]-β-D-Xyl*p*-*O*-Me (X4[X2]XMe; compound **16**)), β-D-Xyl*p*-1,4-[β-DXyl*p*-1,3]-β-D-Xyl*p*-*O*-Me (X4[X3]XMe; compound **18**)), β-D-Xyl*p*-1,3-β-D-Xyl*p*-1,4-β-D-Xyl*p*-*O*-Me (X3X4XMe; compound **26**)), α-DXyl*p*-1,3-β-D-Xyl*p*-1,4-β-D-Xyl*p*-*O*-Me (αX3X4XMe; compound **27**)), α-D-Xyl*p*-1,4-β-D-Xyl*p*-1,3-β-D-Xyl*p*-*O*-Me (αX4X3XMe; compound **19**))) α-D-Xyl*p*-1,4-β-D-Xyl*p*-1,2-β-D-Xyl*p*-*O*-Me (αX4X2XMe; compound **17**))—were synthesized previously [20,21,22,23,24,25] and were generously supplied by Dr. Ján Hirsch (Institute of Chemistry, Slovak Academy of Sciences, Bratislava, Slovakia).

### 4.3. Determination of pH and Temperature Optimum and Temperature Stability

pH optimum was determined at 40 °C using a 15-min incubation of 69 nM *Tl*Xyn30A with 1% solution of GX in 40 mM Britton-Robinson (pH 3.5–5.5) or 50 mM sodium phosphate buffers (pH 6.0–8.0). The optimum temperature was determined in the same way in 40 mM Britton-Robinson buffer, pH 3.5, and temperatures ranging from 23 to 90 °C. Temperature stability was tested in 40 mM Britton-Robinson buffer, pH 3.5, at 40–60 °C for up to 5 h. During the incubation, aliquots were taken at different time points and the residual activity was immediately determined as described above (1% GX, pH 3.5, 40 °C, 15 min).

### 4.4. Hydrolysis of Polysaccharides and Oligosaccharides

Specific activity on polysaccharides was determined by the Somogyi–Nelson procedure [26] as the amount of generated reducing sugars. GX, AX, and Rho solution (1%; *w*/*v*) were incubated at 37 °C with *Tl*Xyn30A (34 nM) in 50 mM sodium acetate buffer, pH 3.5. At time intervals, 100-μL aliquots were taken for the determination of reducing sugars. One unit of enzyme activity was defined as the enzyme amount liberating in 1 min 1 μmol of reducing sugars expressed as an equivalent of xylose.

For TLC (thin-layer chromatography) analysis, the polysaccharide solutions (1% GX, Rho, AraX) were incubated with 0.45 μM *Tl*Xyn30A at 40 °C. After 2 h, a fresh enzyme was added to a final concentration of 2.11 μM. During the reaction course, aliquots of 5 μL were spotted on silica gel-coated aluminum sheets (Merck, Darmstadt, Germany). After 24 h, the reaction was terminated by heating at 100 °C for 5 min. Subsequent treatment with β-xylosidase (1 U/mL) was done overnight at 40 °C. Low-molecular-weight substrates, i.e. linear xylooligosaccharides (Xyl_2_–Xyl_6_), aldouronic acids MeGlcA^3^Xyl_3_ and MeGlcA^3^Xyl_4_, arabinoxylooligosaccharides, methyl and 4-nitrophenyl glycosides, were used at 2.5 mM. Their hydrolysis with 6.25 μM *Tl*Xyn30A was conducted in 50 mM sodium acetate buffer, pH 3.5, at 35 °C, and 2-μL aliquots were spotted on the TLC plate. The TLC plate was developed either twice in a solvent system of ethyl acetate/acetic acid/2-propanol/formic acid/water 25:10:5:1:15 (*v*/*v*) (polysaccharides, aldouronic acids, and linear xylooligosaccharides) or once in a solvent system of *n*-butanol/ethanol/water 10:8:5 (*v*/*v*), and the sugars were visualized with the orcinol reagent.

### 4.5. MALDI-ToF MS

The hydrolysates were decationized by Dowex 50 (H^+^ form) and 1 µL was mixed with 1 µL of the matrix (1% solution of 2,5-dihydroxybenzoic acid in 30% acetonitrile) directly on the MS target plate. After air-drying, the samples were analyzed by a UltrafleXtreme MALDI ToF/ToF mass spectrometer (Bruker Daltonics, Bremen, Germany) operating in reflectron positive mode.

## 5. Conclusions

Although *T. leycettanus* GH30_7 xylanase behaves as a typical endo-β-1,4-xylanase, its catalytic properties are unique in comparison with other types of non-specific endoxylanases. The enzyme hydrolyzes all types of xylan to a higher degree than GH10, GH11, and other GH30 xylanases. The shortest and most abundant fragment yielded by *Tl*Xyn30A from glucuronoxylan (MeGlcA^2^Xyl_2_) is smaller by one and two xylosyl residues than the shortest aldouronic acids generated by GH10 and GH11 xylanases, respectively. Unbranched regions of the polysaccharides and larger oligosaccharides as well as linear β-1,4-xylooligosaccharides are degraded mainly to Xyl_2_. This is important in view of possible biotechnological applications of the enzyme.

## Data Availability

The data presented in this study are available on request from the corresponding author.

## Supplementary Materials

The following are available online. Figure S1: pH and temperature optimum and temperature stability of *Tl*Xyn30A. Figure S2: Multiple sequence alignment of GH30 catalytic domain of *Tl*Xyn30A and selected GH30 xylanases.

## Sample Availability

Samples of the compounds are available from the authors.
