# Supplementary material for: Non-Specific GH30_7 Endo-β-1,4-xylanase from Talaromyces leycettanus
Source: Molecules. 2021 Jul 30;26(15):4614. doi: 10.3390/molecules26154614 (PMC8347862; doi:10.3390/molecules26154614)
Supplement: Supplementary file 1 [file molecules-26-04614-s001.zip › molecules-1300024-supplementary.pdf]

Supplementary material to the manuscript

Non-specific GH30_7 endo-β-1,4-xylanase from *Talaromyces leycettanus*

authored by

Katarína Šuchová, Nikolaj Spodsberg, Kristian B. R. Mørkeberg Krogh, Peter Biely and Vladimír Puchart

A
B
C

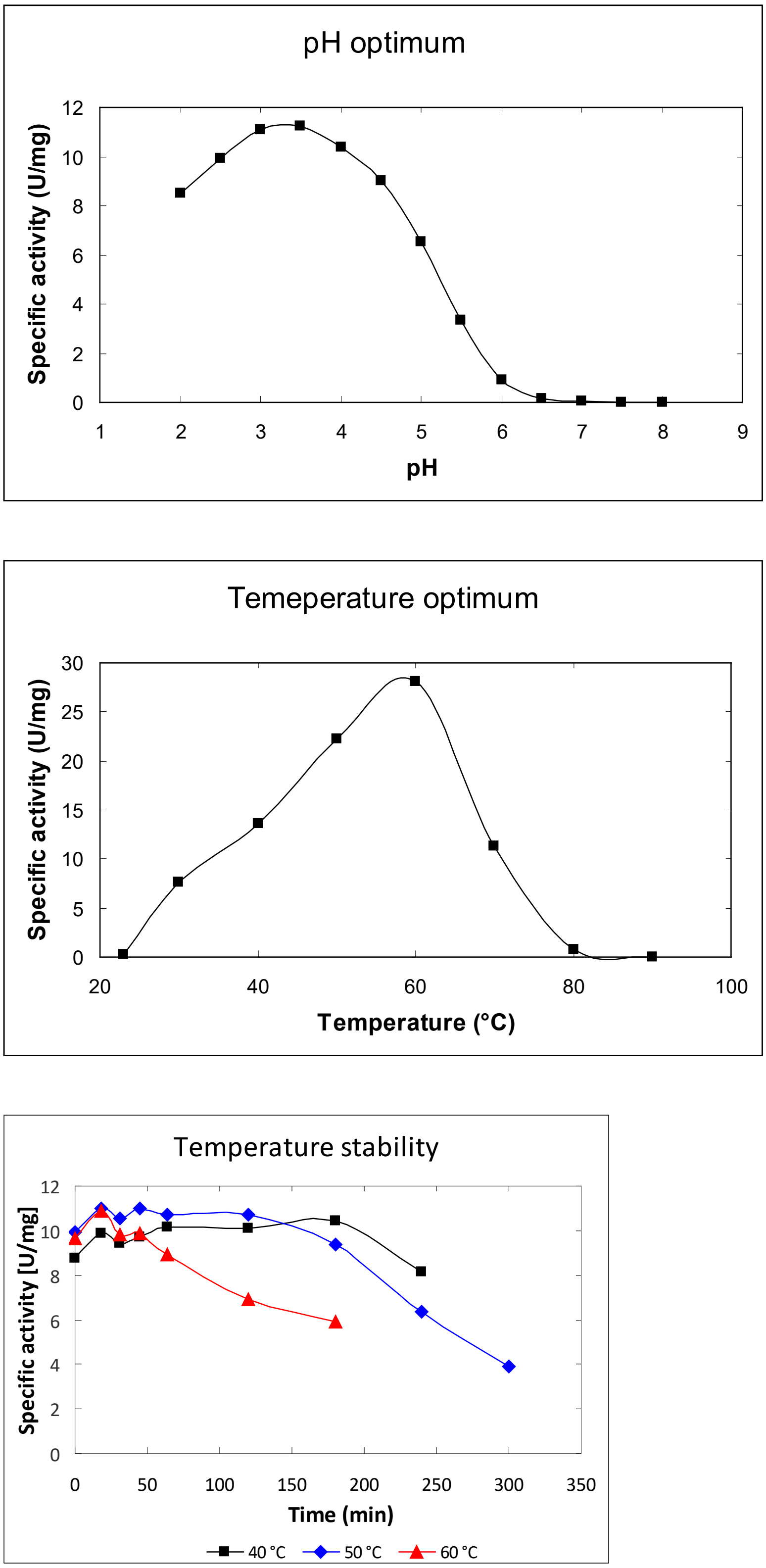

Figure S1. pH and temperature optimum and temperature stability of *Tl*Xyn30A. **A**) pH optimum was determined at 40 °C using a 15 min incubation of the enzyme with 1% GX as a substrate in either 40 mM Britton-Robinson (pH 3.5-5.5) or 50 mM sodium phosphate buffers (pH 6.0-8.0).
B) Temperature optimum was determined under the same conditions using 40 mM Britton-Robinson buffer, pH 3.5, and temperatures ranging from 23 °C to 90 °C. **C**) Temperature stability was tested as follows: the enzyme was heated at 40, 50 or 60 °C for up to 5 hours in 40 mM Britton-Robinson buffer, pH 3.5 (in the absence of the substrate), and then assayed at 40 °C.

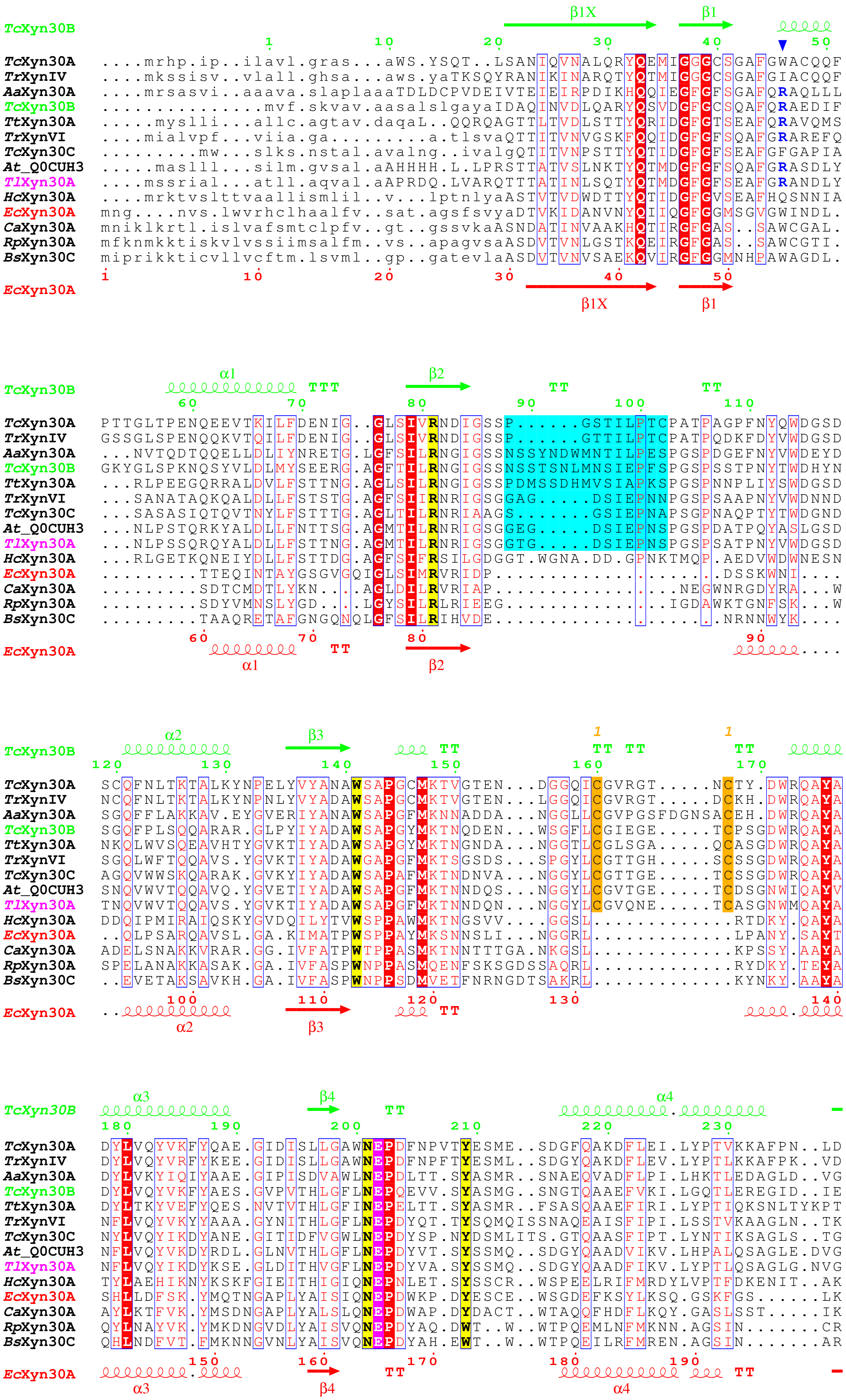

TcXyn30B β1X β1
1 10 20 30 40 50
TcXyn30A ....mrhp.ip..ilavl.gras...aWS.YSQT..LSANIQVNALQRYQEMIGGGCSGAFGWACQQF
TrXynIV ....mkssisv..vlall.ghsa...aws.yaTKSQYRANIKINARQTYQTMIGGGCSGAFGIACQQF
AaXyn30A ....mrsasvi..aaava.slaplaaaTDLDCPVDEIVTEIEIRPDIKHQQIEGFGFSGAFQRAQLLL
TcXyn30B ....mvf.skvav.aasalslgayaIDAQINVDLQARYQSVDGFGCSQAFQRAEDIF
TtXyn30A ....myslli...allc.agtav.daqaL..QQRQAGTTLTVDLSTTYQRIDGFGTSEAFQRAVQMS
TrXynVI ....mialvpf...viia.ga........a.tlsvaQTTITVNVGSKFQQIDGFGFSQAFGRAREFQ
TcXyn30C ....mw...slks.nstal.avalng..ivalgQTITVNPSTTYQTIDGFGFSEAFGFGAPIA
At_Q0CUH3 ....maslll...silm.gvsal.aAHHHH.L.LPRSTTATVSLNKTYQTMDGFGFSQAFGRASDLY
TlXyn30A ....mssrial...atll.aqval.aAPRDQ.LVARQTTTATINLSQTYQTMDGFGFSEAFGRANDLY
HcXyn30A ....mrktvslttvaallismlil..v...lptnlyaASTVTVDWDTTYQTIDGFGVSEAFHQSNNIA
EcXyn30A mng....nvs.lwvrhclhaalfv..sat.agsfsvyaDTVKIDANVNYQIIQGFGGMSGVGWINDL.
CaXyn30A mniklkrtl.islvafsmtclpfv..gt..gssvkaASNDATINVAAKHQTIRGFGAS..SAWCGAL.
RpXyn30A mfknmkktiskvlvssiimsalfm..vs..apagvsaASDVTVNLGSTKQEIRGFGAS..SAWCGTI.
BsXyn30C miprikkticvllvcftm.lsvml..gp..gatevlaASDVTVNVSAEKQVIRGFGGMNHPAWAGDL.
1 10 20 30 40 50
EcXyn30A β1X β1

TcXyn30B α1 TTT β2 TT TT
60 70 80 90 100 110
TcXyn30A PTTGLTPENQEEVTKILFDENIG..GLSIVRNDIGSSP......GSTILPTCPATPAGPFNYQWDGSD
TrXynIV GSSGLSPENQQKVTQILFDENIG..GLSIVRNDIGSSP......GTTILPTCPATPQDKFDYVWDGSD
AaXyn30A ...NVTQDTQQELLDLIYNRETG.LGFSILRNGIGSSNSSYNDWMNTILPESPGSPDGEFNYVWDEYD
TcXyn30B GKYGLSPKNQSYVLDLMYSEERG.AGFTILRNGIGSSNSSTSNLMNSIEPFSPGSPSSTPNYTWDHYN
TtXyn30A ...RLPEEGQRRALDVLFSTTNG.AGLSILRNGIGSSPDMSSDHMVSIAPKSPGSPNNPLIYSWDGSD
TrXynVI ...SANATAQKQALDLLFSTSTG.AGFSIIRNRIGSGGAG....DSIEPNNPGSPSAAPNYVWDNND
TcXyn30C ...SASASIQTQVTNYLFSTTTG.AGLTILRNRIAAGS......GSIEPNAPSGPNAQPTYTWDGND
At_Q0CUH3 ...NLPSTQRKYALDLLFNTTSG.AGMTILRNRIGSGGEG....DSIEPNSPGSPDATPQYASLGSD
TlXyn30A ...NLPSSQRQYALDLLFSTTNG.AGMTILRNRIGSGGTG....DSIEPNSPGSPSATPNYVWDGSD
HcXyn30A ...RLGETKQNEIYDLLFSTTDG.AGFSIFRSILGDGGT.WGNA.DD.GPNKTMQP.AEDVWDWNESN
EcXyn30A .........TTEQINTAYGSGVGQIGLSIMRVRIDP....................DSSKWNI....
CaXyn30A .........SDTCMDTLYKN....AGLDILRVRIAP..................NEGWNRGDYRA...W
RpXyn30A .........SDYVMNSLYGD....LGYSILRLRIEEG.................IGDAWKTGNFSK...W
BsXyn30C .........TAAQRETAFGNGQNQLGFSILRIHVDE...................NRNNWYK....
60 70 80 90
EcXyn30A α1 TT β2

TcXyn30B α2 β3 TT 1 TT TT 1 TT
120 130 140 150 160 170
TcXyn30A SCQFNLTKTALKYNPELYVYANAWSAPGCMKTVGTEN...DGGQICGVRGT.....NCTY.DWRQAYA
TrXynIV NCQFNLTKTALKYNPNLYVYADAWSAPGCMKTVGTEN...LGGQICGVRGT.....DCKH.DWRQAYA
AaXyn30A SGQFFLAKKAV.EYGVERIYANAWSAPGFMKNNADDA...NGGLLCGVPGSFDGNSACEH.DWRQAYA
TcXyn30B SGQFPLSQQARAR.GLPYIYADAWSAPGYMKTNQDEN...WSGFLCGIEGE.....TCPSGDWRQAYA
TtXyn30A NKQLWVSQEAVHTYGVKTIYADAWSAPGYMKTNGNDA...NGGTLCGLSGA.....QCASGDWRQAYA
TrXynVI SGQLWFTQQAVS.YGVKTIYADAWGAPGFMKTSGSDS...SPGYLCGTTGH.....SCSSGDWRQAYA
TcXyn30C AGQVWWSKQARAK.GVKYIYADAWSAPAFMKTNDNVA...NGGYLCGTTGE.....TCSSGDWRQAYA
At_Q0CUH3 SNQVWVTQQAVQ.YGVETIYADAWSAPGFMKTNNDQS...NGGYLCGVTGE.....TCDSGNWIQAYV
TlXyn30A TNQVWVTQQAVS.YGVKTIYADAWSAPGFMKTNNDQN...NGGYLCGVQNE.....TCASGNWMQAYA
HcXyn30A DDQIPMIRAIQSKYGVDQILYTVWSPPAWMKTNGSVV....GGSL.............RTDKY.QAYA
EcXyn30A ..QLPSARQAVSL.GA.KIMATPWSPPAYMKSNNSLI...NGGRL.............LPANY.SAYT
CaXyn30A ADELSNAKKVRAR.GG.IVFATPWTPPASMKTNNTTTGA.NKGSL.............KPSSY.AAYA
RpXyn30A SPELANAKKASAK.GA.IVFASPWNPPASMQENFSKSGDSSAQRL.............RYDKY.TEYA
BsXyn30C ..EVETAKSAVKH.GA.IVFASPWNPPSDMVETFNRNGDTSAKRL.............KYNKY.AAYA
100 110 120 130 140
EcXyn30A α2 β3 TT

TcXyn30B α3 β4 TT α4
180 190 200 210 220 230
TcXyn30A DYLVQYVKFYQAE.GIDISLLGAWNEPDFNPVTYESME..SDGFQAKDFLEI.LYPTVKKAFPN..LD
TrXynIV DYLVQYVRFYKEE.GIDISLLGAWNEPDFNPFTYESML..SDGYQAKDFLEV.LYPTLKKAFPK..VD
AaXyn30A DYLVKYIQIYAAE.GIPISDVAWLNEPDLTT.SYASMR..SNAEQVADFLPI.LHKTLEDAGLD..VG
TcXyn30B DYLVQYVKFYAES.GVPVTHLGFLNEPQEVV.SYASMG..SNGTQAAEFVKI.LGQTLEREGID..IE
TtXyn30A DYLTKYVEFYQES.NVTVTHLGFINEPELTT.SYASMR..FSASQAAEFIRI.LYPTIQKSNLTYKPT
TrXynVI NFLVQYVKYYAAA.GYNITHLGFLNEPDYQT.TYSQMQISSNAQEAISFIPI.LSSTVKAAGLN..TK
TcXyn30C NYLVQYIKDYANE.GITIDFVGWLNEPDYSP.NYDSMLITS.GTQAASFIPT.LYNTIKSAGLS..TG
At_Q0CUH3 NFLVQYVKDYRDL.GLNVTHLGFLNEPDYVT.SYSSMQ..SDGYQAADVIKV.LHPALQSAGLE.DVG
TlXyn30A NFLVQYIKDYKSE.GLDITHVGFLNEPDYVA.SYSSMQ..SDGYQAADFIKV.LYPTLQSAGLG.NVG
HcXyn30A TYLAEHIKNYKSKFGIEITHIGIQNEPNLET.SYSSCR..WSPEELRIFMRDYLVPTFDKENIT..AK
EcXyn30A SHLLDFSK.YMQTNGAPLYAISIQNEPDWKP.DYESCE..WSGDEFKSYLKSQ.GSKFGS......LK
CaXyn30A AYLKTFVK.YMSDNGAPLYALSLQNEPDWAP.DYDACT..WTAQQFHDFLKQY.GASLSST.....IK
RpXyn30A QYLNAYVK.YMKDNGVDLYAISVQNEPDYAQ.DWT..W..WTPQEMLNFMKNN.AGSIN.......CR
BsXyn30C QHLNDFVT.FMKNNGVNLYAISVQNEPDYAH.EWT..W..WTPQEILRFMREN.AGSIN.......AR
150 160 170 180 190
EcXyn30A α3 β4 TT α4 TT

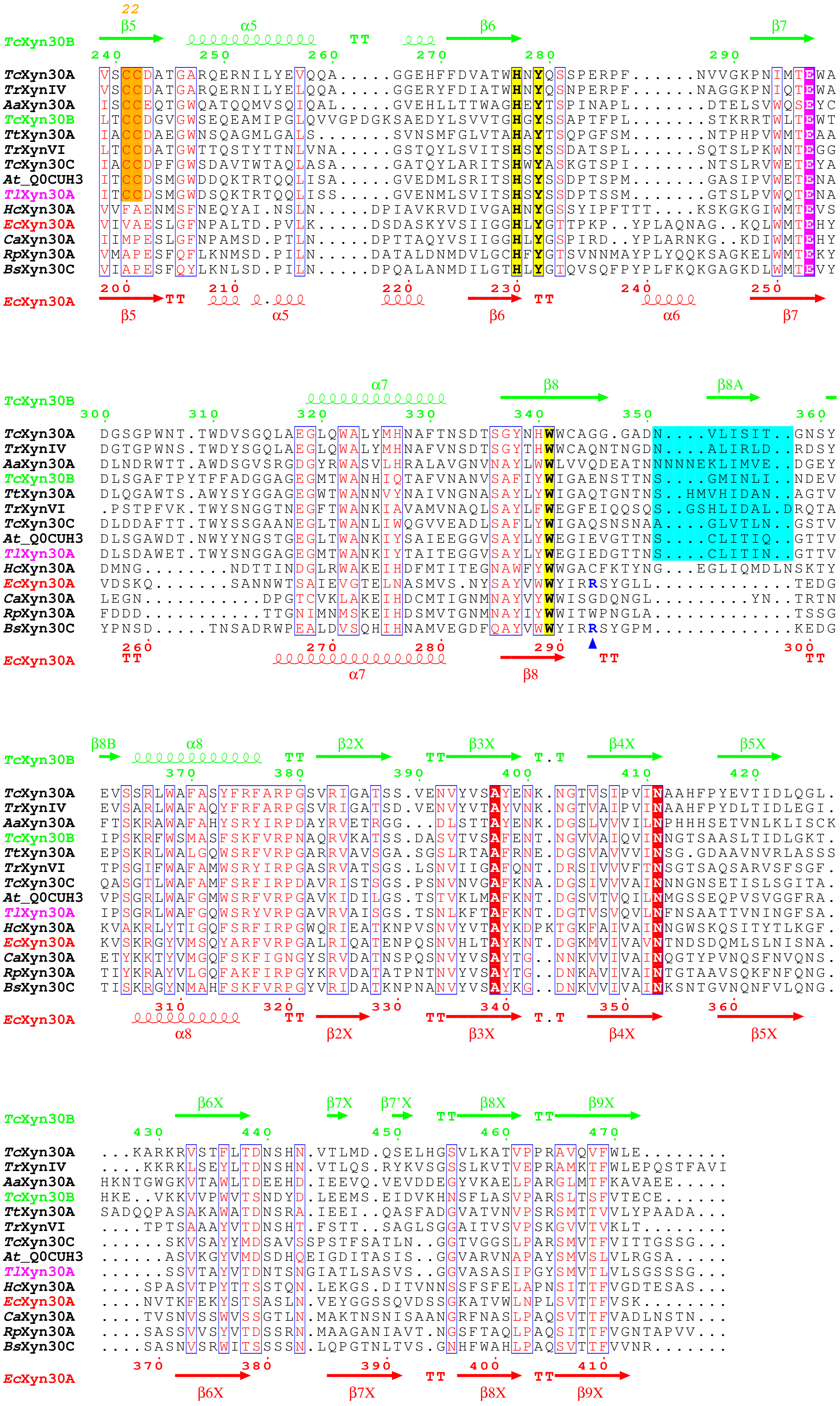
22
TcXyn30B β5 α5 TT β6 β7
240 250 260 270 280 290
TcXyn30A VSCCDATGARQERNILYEVQQA.....GGEHFFDVATWHNYQSSPERPF......NVVGKPNIMTEWA
TrXynIV VSCCDATGARQERNILYELQQA.....GGERYFDIATWHNYQSNPERPF......NAGGKPNIQTEWA
AaXyn30A ISCCEQTGWQATQQMVSQIQAL.....GVEHLLTTWAGHEYTSPINAPL.......DTELSVWQSEYC
TcXyn30B LTCCDGVGWSEQEAMIPGLQVVGPDGKSAEDYLSVVTGHGYSSAPTFPL.......STKRRTWLTEWT
TtXyn30A IACCDAEGWNSQAGMLGALS.......SVNSMFGLVTAHAYTSQPGFSM.......NTPHPVWMTEAA
TrXynVI LTCCDATGWTTQSTYTTNLVNA.....GSTQYLSVITSHSYSSDATSPL......SQTSLPKWNTEGG
TcXyn30C IACCDPFGWSDAVTWTAQLASA.....GATQYLARITSHWYASKGTSPI......NTSLRVWETEYA
At_Q0CUH3 ITCCDSMGWDDQKTRTQQLISA.....GVEDMLSRITSHSYSSDPTSPM.......GASIPVWETENA
TlXyn30A ITCCDSMGWDSQKTRTQQLISS.....GVENMLSVITSHSYSSDPTSSM.......GTSLPVWQTENA
HcXyn30A VVFAENMSFNEQYAI.NSLN.....DPIAVKRVDIVGAHNYGSSYIPFTTT....KSKGKGIWMTEVS
EcXyn30A VIVAESLGFNPALTD.PVLK.....DSDASKYVSIIGGHLYGTTPKP..YPLAQNAG..KQLWMTEHY
CaXyn30A IIMPESLGFNPAMSD.PTLN.....DPTTAQYVSIIGGHLYGSPIRD..YPLARNKG..KDIWMTEHY
RpXyn30A VMAPESFQFLKNMSD.PILN.....DATALDNMDVLGCHFYGTSVNNMAYPLYQQKSAGKELWMTEKY
BsXyn30C VIAPESFQYLKNLSD.PILN.....DPQALANMDILGTHLYGTQVSQFPYPLFKQKGAGKDLWMTEVY
200 210 220 230 240 250
EcXyn30A TT TT
β5 α5 β6 α6 β7
TcXyn30B α7 β8 β8A
300 310 320 330 340 350 360
TcXyn30A DGSGPWNT.TWDVSGQLAEGLQWALYMHNAFTNSDTSGYNHWWCAGG.GADN....VLISIT..GNSY
TrXynIV DGTGPWNS.TWDYSGQLAEGLQWALYMHNAFVNSDTSGYTHWWCAQNTNGDN....ALIRLD..RDSY
AaXyn30A DLNDRWTT.AWDSGVSRGDGYRWASVLHRALAVGNVNAYLWWLVVQDEATNNNNNEKLIMVE..DGEY
TcXyn30B DLSGAFTPYTFFADGGAGEGMTWANHIQTAFVNANVSAFIYWIGAENSTTNS....GMINLI..NDEV
TtXyn30A DLQGAWTS.AWYSYGGAGEGWTWANNVYNAIVNGNASAYLYWIGAQTGNTNS..HMVHIDAN..AGTV
TrXynVI .PSTPFVK.TWYSNGGTNEGFTWANKIAVAMVNAQLSAYLFWEGFEIQQSQS..GSHLIDAL.DRQTA
TcXyn30C DLDDAFTT.TWYSSGAANEGLTWANLIWQGVVEADLSAFLYWIGAQSNSNAA....GLVTLN..GSTV
At_Q0CUH3 DLSGAWDT.NWYYNGSTGEGLTWANKIYSAIEEGGVSAYLYWEGIEVGTTNS....CLITIQ..GTTV
TlXyn30A DLSDAWET.TWYSNGGAGEGMTWANKIYTAITEGGVSAYLYWEGIEDGTTNS....CLITIN..GTTV
HcXyn30A DMNG........NDTTINDGLRWAKEIHDFMTITEGNAWFYWWGACFKTYNG...EGLIQMDLNSKTY
EcXyn30A VDSKQ........SANNWTSAIEVGTELNASMVS.NYSAYVWWYIRRSYGLL.............TEDG
CaXyn30A LEGN...........DPGTCVKLAKEIHDCMTIGNMNAYVYWWISGDQNGL.........YN..TRTN
RpXyn30A FDDD...........TTGNIMNMSKEIHDSMVTGNMNAYIYWWITWPNGLA.............TSSG
BsXyn30C YPNSD.....TNSADRWPEALDVSQHIHNAMVEGDFQAYVWWYIRRSYGPM.............KEDG
260 270 280 290 300
EcXyn30A TT TT TT
α7 β8
TcXyn30B β8B α8 TT β2X TT β3X T.T β4X β5X
370 380 390 400 410 420
TcXyn30A EVSSRLWAFASYFRFARPGSVRIGATSS.VENVYVSAYENK.NGTVSIPVINAAHFPYEVTIDLQGL.
TrXynIV EVSARLWAFAQYFRFARPGSVRIGATSD.VENVYVTAYVNK.NGTVAIPVINAAHFPYDLTIDLEGI.
AaXyn30A FTSKRAWAFAHYSRYIRPDAYRVETRGG...DLSTTAYENK.DGSLVVVILNPHHHSETVNLKLISCK
TcXyn30B IPSKRFWSMASFSKFVRPNAQRVKATSS.DASVTVSAFENT.NGVVAIQVINNGTSAASLTIDLGKT.
TtXyn30A EPSKRLWALGQWSRFVRPGARRVAVSGA.SGSLRTAAFRNE.DGSVAVVVINSG.GDAAVNVRLASSS
TrXynVI TPSGIFWAFAMWSRYIRPGASRVATSGS.LSNVIIGAFQNT.DRSIVVVFTNSGTSAQSARVSFSGF.
TcXyn30C QASGTLWAFAMFSRFIRPDAVRISTSGS.PSNVNVGAFKNA.DGSIVVVAINNNGNSETISLSGITA.
At_Q0CUH3 VPSGRLWAFGMWSRFVRPGAVKIDILGS.TSTVKLMAFKNT.DGSVTVQILNMGSSEQPVSVGGFRA.
TlXyn30A IPSGRLWAFGQWSRYVRPGAVRVAISGS.TSNLKFTAFKNT.DGTVSVQVLNFNSAATTVNINGFSA.
HcXyn30A KVAKRLYTIGQFSRFIRPGWQRIEATKNPVSNVYVTAYKDPKTGKFAIVAINNGWSKQSITYTLKGF.
EcXyn30A KVSKRGYVMSQYARFVRPGALRIQATENPQSNVHLTAYKNT.DGKMVIVAVNTNDSDQMLSLNISNA.
CaXyn30A ETYKKTYVMGQFSKFIGNGYSRVDATNSPQSNVYVSAYTG..NNKVVIVAINQGTYPVNQSFNVQNS.
RpXyn30A TIYKRAYVLGQFAKFIRPGYKRVDATATPNTNVYVSAYTG..DNKAVIVAINTGTAAVSQKFNFQNG.
BsXyn30C TISKRGYNMAHFSKFVRPGYVRIDATKNPNANVYVSAYKG..DNKVVIVAINKSNTGVNQNFVLQNG.
310 320 330 340 350 360
EcXyn30A TT TT T.T
α8 β2X β3X β4X β5X
TcXyn30B β6X β7X β7'X TT β8X TT β9X
430 440 450 460 470
TcXyn30A ...KARKRVSTFLTDNSHN.VTLMD.QSELHGSVLKATVPPRAVQVFWLE........
TrXynIV ....KKRKLSEYLTDNSHN.VTLQS.RYKVSGSSLKVTVEPRAMKTFWLEPQSTFAVI
AaXyn30A HKNTGWGKVTAWLTDEEHD.IEEVQ.VEVDDEGYVKAELPARGLMTFKAVAEE.....
TcXyn30B HKE..VKKVVPWVTSNDYD.LEEMS.EIDVKHNSFLASVPARSLTSFVTECE......
TtXyn30A SADQQPASAKAWATDNSRA.IEEI..QASFADGVATVNVPSRSMTTVVLYPAADA...
TrXynVI ....TPTSAAAYVTDNSHT.FSTT..SAGLSGGAITVSVPSKGVTVKLT........
TcXyn30C ......SKVSAYYMDSAVSSPSTFSATLN..GGTVGGSLPARSMVTFVITTGSSG...
At_Q0CUH3 ......ASVKGYVMDSDHQEIGDITASIS..GGVARVNAPAYSMVSLVLRGSA.....
TlXyn30A ......SSVTAYVTDNTSNGIATLSASVS..GGVASASIPGYSMVTLVLSGSSSG...
HcXyn30A ....SPASVTPYTTSSTQN.LEKGS.DITVNNSSFSFELAPNSITTFVGDTESAS...
EcXyn30A ....NVTKFEKYSTSASLN.VEYGGSSQVDSSGKATVWLNPLSVTTFVSK........
CaXyn30A ....TVSNVSSWVSSGTLN.MAKTNSNISAANGRFNASLPAQSVTTFVADLNSTN...
RpXyn30A ....SASSVVSYVTDSSRN.MAAGANIAVT.NGSFTAQLPAQSITTFVGNTAPVV...
BsXyn30C ....SASNVSRWITSSSSN.LQPGTNLTVS.GNHFWAHLPAQSVTTFVVNR.......
370 380 390 400 410
EcXyn30A TT TT
β6X β7X β8X β9X

Figure S2. Multiple sequence alignment of GH30 catalytic domain of *Tl*Xyn30A and selected GH30 xylanases. The first two sequences, *Tc*Xyn30A (Uniprot ID: A0A0B8MZ29) and *Tr*XynIV (A0A024RWW9), represent reducing-end xylose releasing enzymes. For next three sequences, *Aa*Xyn30A [Ref. 5], *Tc*Xyn30B (A0A4V8H018) and *Tt*Xyn30A (G2Q1N4), the non-reducing end xylobiohydrolase activity has been demonstrated. The latter two enzymes are specific glucuronoxylanases, similarly to *Tr*XynVI (G0RV92). In contrast, *Tc*Xyn30C (A0A6N4SL16) is a non-specific endoxylanase. The next sequence, *At*_QUCUH3, corresponding to an uncharacterized *Aspergillus terreus* protein, shows the highest similarity to *Tl*Xyn30A. The last 5 sequences are of bacterial origin and belong to GH30_8 subfamily. *Hc*Xyn30A (G8LU16) is a unique prokaryotic non-reducing end xylobiohydrolase. *Ec*Xyn30A (Q46961) and *Bs*XynC (Q45070) are canonical GH30_8 glucuronoxylanases. *Ca*Xyn30A (Q97TI2) and *Rp*Xyn30A (F1TBY8) are exceptional GH30_8 non-specific endoxylanases. Secondary structure elements identified in the structures of *Tc*Xyn30B (green) and *Ec*Xyn30A (red) are shown on top and bottom. Catalytic acid and catalytic base are typed on violet background. Another amino acids that are invariant in GH30 xylanases are typed on yellow background. Cyan-highlighted are β2-α2 loop and β8-α8 region (both are shown in detail in **Figure 6**) where there are the greatest differences between GH30_8 and GH30_7 members. Prokaryotic arginine typed in blue and marked at the bottom by an up triangle is responsible for glucuronoxylanases specificity of the bacterial glucuronoxylanases. Eukaryotic arginine, which is believed to contribute to the MeGlcA moiety recognition by the eukaryotic xylanases and is rather well conserved in the GH30_7 enzymes, is also found in *Tl*Xyn30A and is marked on the top by a down triangle Four cysteín residues conserved in the eukaryotic enzymes form a pair of disulphide bonds.
